# Cellulose‐Based Transparent Edible Antibacterial Oxygen‐Barrier Coating for Long‐Term Fruit Preservation

**DOI:** 10.1002/advs.202409560

**Published:** 2024-11-13

**Authors:** Yuqian Cui, Yixiu Cheng, Zhan Xu, Bingchun Li, Weiguo Tian, Jun Zhang

**Affiliations:** ^1^ Beijing National Laboratory for Molecular Sciences CAS Key Laboratory of Engineering Plastics Institute of Chemistry Chinese Academy of Sciences (CAS) Beijing 100190 China; ^2^ University of Chinese Academy of Sciences Beijing 100049 China

**Keywords:** antibacterial, cellulose, edible coating, fruit preservation, oxygen‐barrier

## Abstract

Long‐term preservation of fresh fruit and vegetables without a cold chain is a great challenge to food security because fruits and vegetables are highly vulnerable to poor storage conditions. Fruit spoilage is a complex biochemical process that involves many factors, including microbial reproduction, oxidation, metabolism, and H_2_O evaporation. Only the synergy of the multiple spoilage inhibition methods can achieve long‐term freshness preservation. Herein, a multifunctional cellulose‐based preservation coating with antibacterial, oxygen/water vapor barrier, and antioxidant properties is proposed, which is based on cellulose microgel (CMG) and prepared using multi‐component composites with montmorillonite (MMT), cationic cellulose derivative (Cell‐P^+^), and L‐ascorbic acid (Vc). It has good wetting properties on fruits with different surfaces. This method can successfully preserve the long‐term freshness of various fruits. This highly transparent, edible, and washable multifunctional cellulose‐based fruit preservation coating can improve the quality of agricultural products, extend the shelf life of food, and reduce the cost of cold‐chain transportation.

## Graphical abstract

A multifunctional cellulose‐based preservation coating with antibacterial, oxygen/water vapor barrier, and antioxidant properties, which is based on cellulose microgel (CMG) and prepared using multi‐component composites with montmorillonite (MMT), cationic cellulose derivative (Cell‐P^+^), and L‐ascorbic acid (Vc). It has good wetting properties, highly transparent, edible, and washable. This method can successfully preserve the long‐term freshness of various fruits.

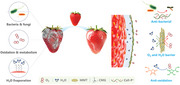

## 1 Introduction

The food crisis remains a worldwide challenge. The COVID‐19 pandemic, climate change, and conflicts have aggravated global food insecurity.^[^ ^1^, ^2^ ^]^ According to the Food and Agriculture Organization of the United Nations (FAO), nearly one‐third of the global food supply is lost or wasted annually. Especially for short‐shelf‐life fruits and vegetables and other easily spoiled foods, the loss ratio is over 40%.^[^ ^3^, ^4^ ^]^ Food preservation technologies that can effectively inhibit spoilage and extend the shelf life of fruit and vegetables are of great significance to improving food security and nutrition, enhancing public health, and alleviating the global food crises.^[^ ^5^, ^6^ ^]^

Food spoilage results from many complex biochemical processes, including microbial reproduction, oxidation, aerobic and anaerobic metabolism, and water evaporation.^[^ ^7^ ^]^ Therefore, the key to long‐term food preservation is inhibiting or blocking the main processes of food spoilage. Antibiotic additives and antioxidant preservatives (e.g., ascorbic acid, citric acid, sodium sorbate, and sorbates) are commonly used to inhibit the growth of microorganisms and oxidative spoilage, respectively, thus prolonging the storage lifetime of food (e.g., juice).^[^ ^8^, ^9^ ^]^ However, excessive use of antibiotic additives and antioxidant preservatives is a new threat to human health. Polymer films and plastic wraps can prevent bacteria and fungi from contaminating food, inhibit oxidative deterioration and aerobic respiration, and reduce water evaporation. This is the most effective and widely used preservation method for fruits and vegetables. However, single‐use, non‐degradable plastic films cause serious microplastic pollution worldwide.^[^ ^10^ ^]^ Other fresh fruit and vegetable preservation technologies include gamma irradiation, which can effectively remove microorganisms on the surface of fruits and vegetables,^[^ ^11^ ^]^ and cold chain, controlled atmosphere, or CO_2_ atmosphere storage, which can inhibit microbial reproduction and respiratory metabolism.^[^ ^12^ ^]^ These methods depend on large facilities, are only suitable for centralized storage and transportation of fruits and vegetables, and are not suitable for preserving fruits and vegetables on shelves for sale. Sustainable solutions are expected to replace plastic wraps to preserve fresh fruits and vegetables.

Barrier coating can also block microorganisms, oxygen, and water evaporation, similar to plastic wrapping. It can adapt to the irregular shape of different fruits and vegetables and directly form an effective microbial and oxygen barrier layer on the surface, which is suitable for preserving fruits and vegetables on the shelf. For instance, fruit waxing is a typical coating technique for fruit preservation using natural wax or artificial wax, which provides a barrier to moisture loss, prevents bruising or other physical damage, and delays the ripening and browning of fruits.^[^ ^13^ ^]^ Therefore, wax‐coated fruit can remain fresh and appealing longer than uncoated fruit. It should be noted that synthetic wax is difficult to clean thoroughly before consumption, and the potential consumption risk of synthetic wax is inevitable.^[^ ^14^ ^]^ In contrast, natural coating materials such as polysaccharides,^[^ ^15^ ^]^ proteins,^[^ ^5^, ^16^ ^]^ lipids,^[^ ^17^ ^]^ chitosan,^[^ ^18^, ^19^ ^]^ and alginate^[^ ^20^ ^]^ are much safer than synthetic wax. Micro/nanocellulose has good film‐forming properties, high mechanical strength, excellent gas barrier properties, and thermal stability, as well as being resourceful, low‐cost, biocompatible, non‐toxic, and edible.^[^ ^21^, ^22^, ^23^, ^24^, ^25^ ^]^ Therefore, it is an ideal candidate material for fruit and vegetable preservation coating.

The conventional preservation coating technology is mainly achieved through physical isolation from the exterior environment. The mechanism complexity of food spoilage determines that only physical isolation is not the best solution for long‐term preservation. Coating materials must be adapted to diverse fruits and vegetables with different shapes and surface properties (wettability and roughness). To avoid changing the appearance of fruits and vegetables on the shelf, next‐generation multifunctional preserving coatings should be transparent, edible, or easily washed off.^[^ ^26^ ^]^ Herein, we propose a multifunctional cellulose‐based preservation coating with antibacterial, oxygen/water vapor barrier, and antioxidant properties and successfully achieve long‐term preservation of perishable fruits. The coating is composed of cellulose microgel (CMG), montmorillonite (MMT), cationic cellulose derivatives (Cell‐P^+^), and L‐ascorbic acid (Vc). Cell‐P^+^ is the main antibacterial component. It can also assist in the delamination of MMT from the bulk material, regulate the wettability of coating emulsions on different fruit surfaces, and promote the spread of the coating lotion. CMG stabilized the MMT nanosheet suspension and increased the mechanical strength of the coating. As the basic gas barrier building block, MMT sheets can be tightly packed on the surface of fruits and vegetables to form an ultrathin coating with good mechanical properties by spraying or dipping owing to the shear‐thinning CMG. Ascorbic acid further supplements antioxidant capacity. The shelf life of strawberries and grapes can be achieved for more than one week by using a preservation coating with the cooperation of antibacterial, oxygen‐blocking, and antioxidant functions. In addition, the coating is transparent, does not affect the appearance of fruits and vegetables for sale, and can be completely washed before eating. Moreover, biological evaluation experiments have shown that coating materials are non‐cytotoxic and can be consumed directly without toxic residues. The advantages of simple preparation, convenient operation, non‐toxicity, and edibility endow the cellulose‐based multifunctional barrier coating material with significant application potential and prospects for the long‐term storage of fruits and vegetables.

## 2 Results and Discussion

### 2.1 Conceptual Design

**Figure 1:**
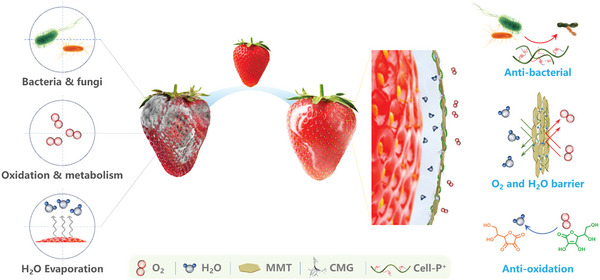
Schematic illustration of cellulose‐based barrier coating for long‐term fruit preservation.

Fruit spoilage is caused by several factors, including microbial growth, oxidation, aerobic and anaerobic metabolism, and water evaporation. To maintain freshness for an extended period, it is essential to inhibit multiple pathways of spoilage. In this study, a multifunctional fruit and vegetable preservation coating material integrating multiple preservation mechanisms, including antimicrobial, oxygen, and water vapor barrier and antioxidant, was designed based on CMG. As illustrated in **Figure** **1**, Cell‐P^+^ is the primary antimicrobial component in the cellulose‐based freshness preservation coating, which can destroy the cell membrane of bacteria through the electrostatic effect. Additionally, Cell‐P^+^ plays a significant role in facilitating the separation of the MMT lamellar structure. The oxygen and water vapor barrier is mainly attributed to the dense membrane formed by CMG and the MMT nanosheets, which can provide a good barrier to oxygen and water vapor. The antioxidant effect is derived from Vc, which can scavenge free radicals and avoid oxidative decay of fruits. CMG is the main film‐forming component, and its shear‐thinning nature can effectively disperse the functional components and maintain long‐term suspension stability, which is conducive to long‐term storage of the coating lotion and spraying of the film. The full synergy between the components forms a dense protective film that is antibacterial, antioxidant, oxygen, and water vapor barrier, which effectively inhibits the fruit from decaying, thus realizing the long‐lasting freshness of the fruit.

### 2.2 Fabrication and Fundamental Properties of the Barrier Coating

**Figure 2:**
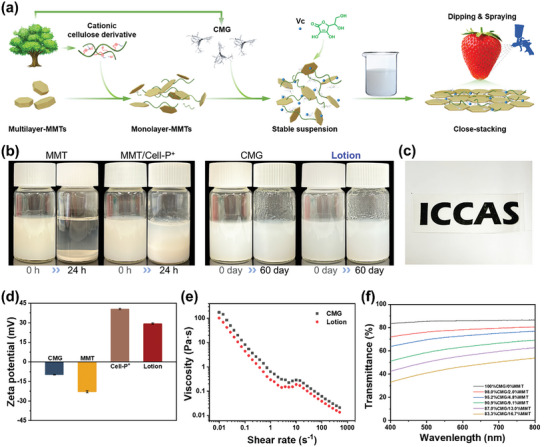
Fundamental properties of the cellulose‐based barrier coating material. (a) Schematic preparation of the cellulose‐based coating material and the coating methods. (b) Suspension stability of the coating lotion and its components after standing for 24 h and 60 days. (c) Photograph of the transparent coating film (thickness, 70 µm). (d) Zeta potential of CMG, MMT, Cell‐P^+^, and the coating lotion. (e) Shear‐thinning viscosity curves of CMG and the coating lotion. (f) Transmittance spectra of the transparent coating films (thickness, 70 µm) with different MMT contents.

The preparation and application of the cellulose‐based multifunctional fruit preservation coating lotion is illustrated in **Figure** **2**a and can be broadly divided into three stages. The first stage involves the synthesis of Cell‐P^+^ (Figures S1 and S2, Supporting Information), the preparation of CMG (Figure S4, Supporting Information), and the exfoliation of MMT into a monolayer structure. Cell‐P^+^ possesses a strong positive charge (as demonstrated in Figure 2d), which provides the essential antibacterial properties of the coating and aids in the electrostatic separation of the MMT into a single monolithic layer. The positively charged Cell‐P^+^ can be intercalated between MMT layers with negative charges (as seen in Figure 2d), resulting in an effective exfoliation of MMT. From the XRD spectra (Figure S5, Supporting Information), it is evident that the incorporation of Cell‐P^+^ decreases the 2*θ* of MMT from 6.08 to 4.90, and the MMT layer spacing increases from 1.45  to 1.80 nm as calculated from the Bragg formula, suggesting that Cell‐P^+^ can be integrated into the interlayer structure of MMT. In addition, Cell‐P^+^ exhibits favorable biodegradability (Figure S3, Supporting Information) and it is unlikely to cause any adverse environmental effects.

In the second stage, a stable monolayer MMT suspension is essential for producing a highly transparent, dense oxygen and water vapor barrier layer. As demonstrated in Figure 2b, the MMT suspension could only be dispersed with the aid of ultrasound, and it was extremely unstable, resulting in noticeable sedimentation and delamination after 24 h at room temperature. Although the dispersion stability of the MMT was improved by ultrasonication assisted by Cell‐P^+^ due to its electrostatic repulsion, the suspension system remained inhomogeneous. However, the addition of CMG significantly enhanced the stability of the coating lotion, and no precipitation was observed after standing for two months, which is extremely beneficial for the subsequent spray or dip coating process on fruit surfaces and long‐term storage of the coating lotion.

In the third stage, the preservation coating for the fruit was prepared by either spraying or dip‐coating. The incorporation of CMG imparted the coating lotion with excellent shear‐thinning properties. As illustrated in Figure 2e, the zero‐shear viscosity of the lotion surpassed 100 Pa·s, facilitating the uniform adherence of the coating lotion to the fruit's surface and the formation of a homogeneous film upon dipping (thickness approx. 5 µm, Figure S6, Supporting Information). Additionally, the viscosity of the lotion decreased rapidly with an increased shear rate by up to four orders of magnitude during spraying. The lower the viscosity, the smaller the size of atomized droplets, resulting in a thinner and more uniform coating on the fruit surface. The application of the coating film resulted in a transparent layer on the fruit surface (Figure 2c) that did not impact the fruit's visual appearance (Figure S10, Supporting Information). The coating film's transmittance is significantly influenced by the MMT content. As shown in Figure 2f, increasing the MMT content from 0% to 16.7 wt.% resulted in a decrease in the transmittance of the self‐standing film (70 µm), particularly for the short‐wavelength transmittance. To maintain the fruit's visual appeal, a 2 wt.% MMT addition is optimal when considering light transmittance alone. However, taking the gas barrier properties and mechanical strength (Figures 4 and 5) of the coating film into account, the preferred MMT addition is set at 4.8 wt.% in the coating lotion.

### 2.3 Biocompatibility, Antibacterial, and Antioxidant Properties of the Barrier Coating

**Figure 3:**
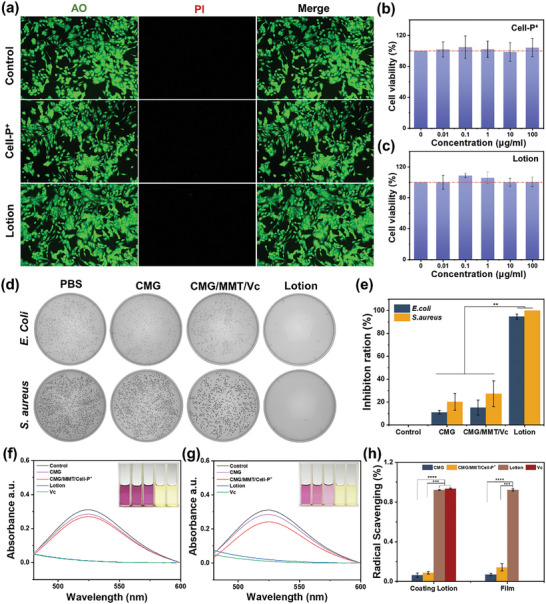
Biocompatibility, antibacterial, and antioxidant properties of cellulose‐based barrier coating. (a) Dual‐fluorescence (AO/PI) images and (b,c) cell viability of GES cells cultured in different concentrations of Cell‐P^+^ and coating lotion, respectively. Using DMEM medium as the positive control (*n* = 4). (d) images of the bacterial colonies and (e) the related inhibition ratios of *E. coli* and *S. aureus* incubated with PBS, CMG, CMG/MMT/VC, coating lotion (1.0 wt.%) in the dark for 30 min (*n* = 3). (f–h) DPPH radical scavenging activity of coating lotion and coating film (*n* = 4).

Good biocompatibility is an essential prerequisite for applying coating lotion in fruit preservation to prevent potential health hazards from residual coating materials due to inadequate cleaning. To evaluate the biosafety of the coating lotion and Cell‐P^+^, a cytotoxicity test is conducted using the human gastric mucosal epithelial cell line (GES). GES cells are co‐cultured with the coating lotion and Cell‐P^+^ at various concentration gradients ranging from 0.01 to 100 µg mL^−1^ for 24 h. The AO/PI results demonstrate that the GES cells maintain high viability (AO) and do not exhibit apoptosis (PI) following 24 h of co‐cultivation with the coating lotion and Cell‐P^+^ (**Figure** **3**a). The CCK‐8 assay indicates that the survival rate of GES cells approximates 100% across different concentrations of coating lotion (Figure 3c) and Cell‐P^+^ (Figure 3b). These assays suggest that the coating lotion and Cell‐P^+^ do not exhibit cytotoxicity toward human GI‐associated cells and demonstrate favorable food safety. This result is consistent with the fact that the ingredients in the coating lotion are all food‐safe.

Antimicrobial preservation coatings are effective in inhibiting microbial growth, thus preventing fruits from decaying and spoiling. The coating lotion that contains Cell‐P^+^ effectively inhibits the growth of colonies, with an inhibition rate of 94.69% for *E. coli* and 100% for *S. aureus*, as shown in Figure 3d,e. In comparison, the CMG inhibited *E. coli* by only 10.87% and *S. aureus* by 20.12%, while the Cell‐P^+^ free coating lotion inhibited *E. coli* by 15.13% and *S. aureus* by 27.00%. The results indicate that the excellent antibacterial properties of the coating originate from the presence of Cell‐P^+^, which binds to the negatively charged bacterial surfaces through electrostatic adsorption, thus destroying the bacterial membrane and leading to the death of the bacteria (Optimal addition of Cell‐P^+^ is illustrated in Figure S7, Supporting Information). The excellent antibacterial activity of the coating reduces the spoilage of the fruit during storage and transportation, prolongs the shelf life, and effectively enhances food safety.

Fruits are a rich source of phenolics that offer numerous health benefits, but when exposed to air, they are prone to oxidation, which inactivates these beneficial compounds and results in a loss of nutrients. Applying an antioxidant coating can effectively prevent oxidative deterioration of fruits. DPPH radical scavenging rate is tested to assess the antioxidant properties of the nanocomposite coating lotion and the coating film. As shown in Figure 3f,g, the Vc and coating lotion (CMG/MMT/Cell‐P^+^/Vc) and coating film demonstrated a strong scavenging effect on DPPH radicals, as evidenced by the discoloration and disappearance of the characteristic UV absorption peak of DPPH at 517 nm. In contrast, the CMG, CMG/MMT/Cell‐P^+^ lotion, and coating film without Vc showed no scavenging effect on DPPH radicals, as the DPPH solution remained unchanged and the characteristic peak at 517 nm remained visible. These results indicate that the addition of Vc imparted excellent antioxidant properties (Optimal addition of Vc is illustrated in Figure S8, Supporting Information) to both the coating lotion and the subsequently formed film, with a free radical scavenging rate of over 90% (Figure 3h), making it an effective means of preventing oxidative spoilage of fruits and vegetables during preservation.

### 2.4 MMT‐Content‐Dependent Gas‐Barrier Properties of the Coating

**Figure 4:**
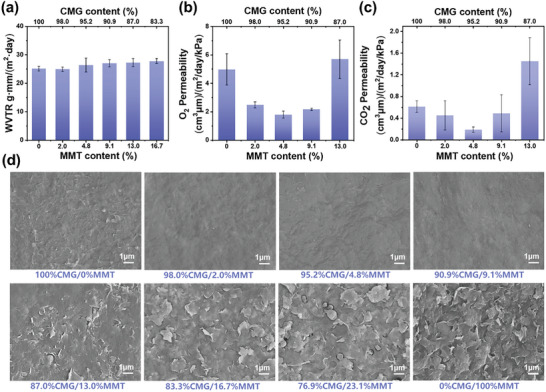
Gas‐barrier properties of the cellulose‐based coating films with different MMT content. (a) Water vapor transmission rate (WVTR), (b) O_2_ permeability, (c) CO_2_ permeability, (d) and surface micromorphology of the coating films with different MMT content (*constant CMG concentration, 0.5* *g*) (*n* = 3).

The water vapor barrier function of the coating material is crucial for preventing fruit dehydration and maintaining its firmness. In addition, the gas (O_2_ and CO_2_) barrier properties of the coating material play a significant role in reducing cell respiration and slowing fruit ripening. Thus, the water vapor transmission rates (WVTR) and gas permeability of the coating material have a direct impact on its preservation capabilities. As seen in **Figure** **4**a–c, the coating material displayed a very low WVTR of less than 30 g·mm/(m^2^·day) at various MMT concentrations. This value is lower than that of other common packaging materials, such as polylactic acid film (174 g·mm/(m^2^·day)), starch film (33 g·mm/(m^2^·day)), and chitosan film (141 g·mm/(m^2^·day)).^[^ ^5^ ^]^ Moreover, the O_2_ and CO_2_ permeabilities of the films with different MMT additions decreased and then increased with the increase in MMT contents. Additionally, the films had the lowest O_2_ and CO_2_ permeabilities at 4.8 wt.% MMT additions, with values of 1.79 cm^3^·µm/(m^2^·24h·KPa) and 0.18 cm^3^·µm/(m^2^·24h·KPa), respectively. The oxygen permeability of our packaging material is significantly lower than that of other common materials, such as polylactic acid film (177 cm^3^·µm/(m^2^·24h·KPa)), starch film (63 cm^3^·µm/(m^2^·24h·KPa)), and chitosan film (19 cm^3^·µm/(m^2^·24h·KPa)).^[^ ^5^ ^]^ The surface micromorphology in Figure 4d gradually becomes smoother as the content of cellulose (CMG) increases because CMG is the primary film‐forming and reinforcement agent in the film, when the montmorillonite (MMT) content is below 9.1 wt.%, the uniform and dense film structure formed by the close packing of monolayer MMT assisted with CMG. However, when the MMT content exceeds 9.1 wt.%, the dense structure is hard to form due to the self‐aggregation of MMT, and the film's surface becomes rough. As demonstrated in Figure 4a, the impact of the mass ratio CMG/MMT on WVTR appears to be minimal. It suggests that the water vapor is mainly diffused across the hydrophilic CMG networks rather than the micropores in the films. In contrast, O_2_ and CO_2_ penetrate the film primarily through the micropores, and the permeability of O_2_ and CO_2_ initially decreases and subsequently increases with the MMT contents, which precisely coincides with the micromorphological changes observed in the SEM images (Figure 4d).

### 2.5 The Mechanical Properties of the Cellulose‐Based Coating Film

**Figure 5:**
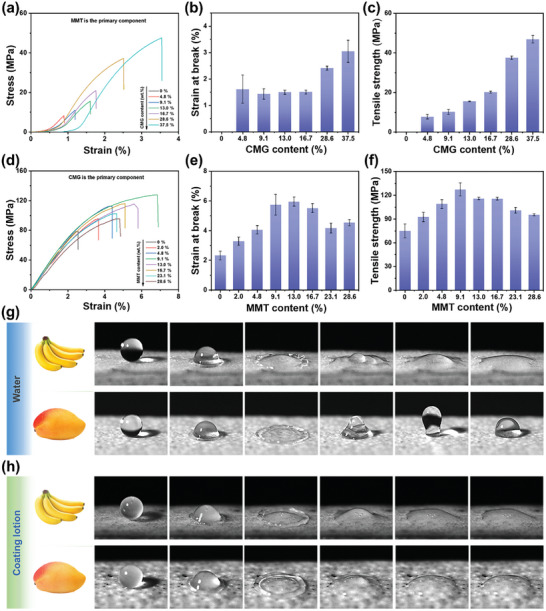
Mechanical properties of the cellulose‐based barrier coating and the spreading behavior of the coating lotion on fruits. (a) The stress–strain curves, (b) strain at break, and (c) tensile strength of the films with different CMG content (*constant MMT content, 0.6* *g*). (d) The stress–strain plots, (e) strain at break, and (f) tensile strength of the film with different MMT content (*constant CMG concentration, 0.5* *g*) (*n* = 5). (g,h) The high‐speed snapshot of water and coating lotion droplets compacting on the waxy surfaces of bananas and mangoes.

The preservation of fruits is highly dependent on the integrity of the cling film layer, which relies on the mechanical properties of the preservation coating film. CMG is crucial for forming the coating film since only MMT suspension cannot produce an intact film (Figure S11, Supporting Information). CMG can also improve the mechanical properties of the film, as demonstrated by the increase in strain at break and tensile strength with the amount of CMG added (**Figure** **5**a–c). However, when CMG was the dominant component, the mechanical properties of the film increased and then decreased with the addition of MMT (Figure 5d–f). The best mechanical properties were observed when the MMT addition was 9.1 wt.%. The microstructure of the film also showed that when the MMT addition was below 9.1 wt.%, the surface of the film was uniform and dense, but as the MMT addition increased, the surface became rough. The internal defects of the material increased, resulting in a decrease in the mechanical properties of the film. The mechanical strength of the cellulose‐based preservation coating film was found to be 104 MPa, comparable to that of traditional PE packaging material (99 MPa). The robustness of the cellulose‐based preservation coating film is expected to significantly extend the durability of the preservation coating in practical applications.

### 2.6 Spreading Behavior of the Coating Lotion on Different Fruit Surfaces

The distribution of the coating lotion on the fruit surface is affected by the lotion's wettability and the fruit surface's hydrophilicity, which in turn influences the spreading behavior of the lotion. As shown in Figure 5g,h, banana, and mango, with distinct surface hydrophilicities (Figure S14, Supporting Information), were selected for the experiments. The results showed that water droplets spread rapidly on the banana's surface. On the mango's waxy surface, the droplet bounced up after spreading and then fell back, hindering the formation of a film during spraying (Figure S15, Supporting Information). In contrast, the coating lotion spread uniformly on both surfaces without any droplet rebound during the spreading process. To explore the effects of different components in improving the spreading effect of the coating lotion on the fruit surface, the spreading of CMG, MMT, Cell‐P^+^, and MMT/Cell‐P^+^ was tested on the fruit surfaces (Figure S16, Supporting Information). The study demonstrated that MMT, Cell‐P^+^, and MMT/Cell‐P^+^ displayed rapid spreading on the banana surface, while the mango surface showed an unusual behavior of droplets bouncing up after spreading and then falling back. This phenomenon is attributed to the comparable viscosity of MMT and Cell‐P^+^ to that of water, resulting in a spreading behavior similar to water. In contrast, CMG droplets exhibited uniform spreading on both banana and mango surfaces due to the increased viscosity of CMG, which enhances the spreading and adhesion of the coating lotion on the waxy fruit surface.^[^ ^27^ ^]^

### 2.7 Performance and Application of the Freshness‐Preserving Coating

**Figure 6:**
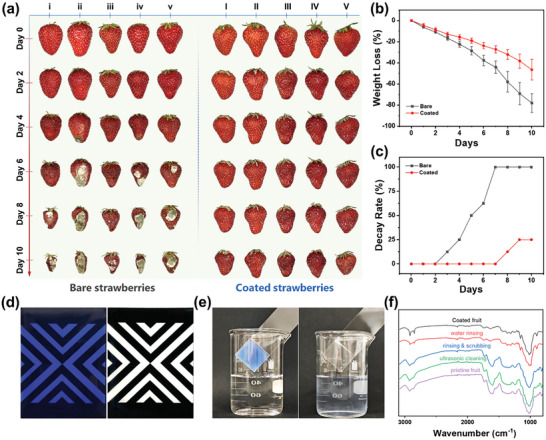
Performance of the cellulose‐based barrier coating for long‐term fruit preservation. (a) Time‐lapse photographs of bare and coated strawberries. (b,c) Weight loss and decay rate of strawberries during storage. (d) Uniform coverage and transparency of coating on the test board. (e) Solubility of the coating film in water (dissolved in 30 s using ultrasonic cleaning). (f) FTIR analysis of the potential residual coating material on fruits (Shine Muscat) washed by different cleaning methods.

The efficacy of cellulose‐based barrier coating in fruit conservation is thoroughly evaluated using non‐climacteric strawberry and climacteric Shine Muscat as examples. As exhibited in **Figure** **6**a, there was no discernible variation in the appearance of the uncoated and coated strawberries on the first day, suggesting that the coating did not affect the appearance of the fruits. The uncoated strawberries began to deteriorate and develop mold on the 3rd day, and by the 7th day, they were all rotten. In contrast, the strawberries coated with the preservation coating showed no signs of decay or molding even after 10 days. This outcome indicated that the preservation coating effectively prevented spoilage brought on by microorganisms. The weight loss data comparison demonstrated that the uncoated strawberries experienced a weight loss rate of 78.11% after 10 days, while the strawberries coated with the preservation compound had a weight loss rate of only 46.54%, as shown in Figure 6b. This indicated that the cellulose‐based barrier coating was capable of retaining the moisture of the fruits to a certain extent, thereby preventing them from dehydration and shriveling.

Similarly, the uncoated Shine Muscat grapes exhibited serious dehydration during storage, and the stalk was partially detached from the flesh (Figure S17a, Supporting Information). As a result, the internal flesh was exposed to air, leading to enzymatic browning and mold growth by the 6th day. By the 10th day, complete spoilage was observed. In contrast, the coated Shine Muscat grapes maintained an intact appearance even after 12 days. Figures S17b and S12c (Supporting Information) illustrate that the uncoated Shine Muscat grapes experienced a 28.52% weight loss after 12 days, whereas the coated Shine Muscat grapes exhibited only a 13.90% weight loss. These results imply that the coating can effectively preserve the fruits, thereby extending their shelf life.

Transparency and uniform coverage of the coating lotion on the fruit surface can be well illustrated through the painting cardboard test in Figure 6d. The cardboard can be fully covered without any defects after applying the lotion dyed with methyl blue, suggesting that the coating can be uniformly applied onto the fruit surface. Moreover, the pattern of undyed coating applied on the cardboard can be clearly seen after it dries to form a film, indicating that the coating possesses excellent transparency and will not affect the aesthetic appeal of the fruit. Figure 6e provides evidence of the cleaning process for the cellulose‐based barrier coating. After 30 s of ultrasonic cleaning in water, the coating (stained) was completely dissolved in the water, which indicates that the coating can be fully removed by washing before consumption.

The coating's adherence and stability under various washing conditions are thoroughly investigated via in situ ATR‐FTIR (Figure 6f) and UV–vis spectroscopic (Figure S18, Supporting Information). A strong peak at 1634 cm^−1^ (stretching vibration of ‐OH on phenolic groups) is observed for the bared fruit due to the abundant phenolic substances in the fruit, and it disappears when the fruit is covered with the coating film. After the coated fruits are subjected to ultrasonic cleaning and manual scrubbing, the characteristic peak of phenolic substances is observed again. Additionally, the characteristic peak assigned to quaternary phosphonate in the coating film ≈2900 cm^−1^ (stretching vibration of C─H on the methyl group in quaternary phosphonate) evidently diminishes. On the contrary, for the coated fruit rinsed with water, the FTIR characteristics are almost identical to those of the coated fruit. The above results demonstrate that the adhesion between the coating film and the fruit is strong enough to prevent water soaking and rinsing. However, the coating film can be effectively removed by ultrasonic or scrubbing methods before consumption.

## 3 Conclusion

In this work, we developed a highly transparent, edible, and removable composite coating material based on CMG to tackle intricate fruit spoilage processes. The experimental results suggested that this coating material exhibited a remarkable 94.69% inhibition for *E. coli* and 100% inhibition for *S. aureus*. Moreover, the water vapor transmission rate (WVTR) of the coating film was found to be lower than 30 g·mm/(m^2^·day). When 4.8 wt.% of MMT was added, the film demonstrated the lowest oxygen and carbon dioxide permeabilities, which were 1.79 cm^3^·µm/ (m^2^·24h·KPa) and 0.18 cm^3^·µm/ (m^2^·24h·KPa), respectively. Additionally, the DPPH free radical scavenging rate was over 90%, and the coating material also exhibited good biocompatibility. Benefiting from the antimicrobial, antioxidant, and water vapor barrier properties, the barrier coating effectively inhibits spoilage and dehydration caused by microbial proliferation, oxidation, aerobic and anaerobic metabolism, and water evaporation. Furthermore, this coating can be effectively applied to fruits with varying surface wettability characteristics, ensuring uniform adhesion to the surface and forming a resilient, transparent film without altering the natural appearance of the fruits. The coated fruits demonstrate reduced weight loss and increased hardness. Such a cellulose‐based barrier coating significantly extends the shelf life of the fruits by ≈ 1 week and shows great potential in fruit preservation.

## 4 Experimental Section

### Materials and Reagents

1‐allyl‐3‐methylimidazolium chloride (AmimCl, water content ≤ 0.3 wt.%) was provided by Shandong Zhongke Henglian Bio‐based Materials Co., LTD. Microcrystalline cellulose (MCC, DP = 220), Tributyl phosphine (TBUP), 2‐chloropropionyl chloride, Dimethylformamide (DMF), Montmorillonite (MMT), L‐ascorbic acid (Vc), 1, 1‐diphenyl‐2‐picrylhydrazyl (DPPH), Methyl blue were commercially available from Innochem (Beijing). CMG was provided by Green Micro&Nano (Beijing, China; www.gmn.com.cn). Phosphate buffered saline (PBS), Dulbecco's modified eagle medium (DMEM), fetal bovine serum (FBS), penicillin/streptomycin solution (P/S), trypsin‐EDTA digestion solution, CCK‐8 kit, and AO/PI staining solution were supplied by Biorigin (Beijing). Bacteria (*E. coli* and *S. aureus*) were purchased from the China General Microbiological Culture Collection Center (CGMCC). Luria‐Bertani (LB) Agar (yeast extract, 5 g; tryptone, 10 g; NaCl, 10 g; agar, 20 g; H_2_O, 1 L) was prepared in the laboratory according to standard procedures. MCC was dried with a vacuum oven at 80 °C for 24 h before use. Other chemical reagents and solvents were used directly without purification.

### Methods—Cell‐P*^+^*

First, 1 g of MCC was added to 24 g of AmimCl (4 wt.%). The mixture was then heated to 80 °C and vigorously stirred under a vacuum until MCC was completely dissolved. Once dissolved, the temperature was lowered to 40 °C, and 3.66 mL of 2‐chloropropionyl chloride was added. The mixture was vigorously stirred for 6 h. Next, the solution was precipitated in 800 mL of ethyl alcohol. The precipitate was then centrifuged and washed with ethyl alcohol three times. Finally, it was dried under a vacuum at 80 °C, producing a white powder (**Cell‐Cl**).

Second, a mixture of 1 g of Cell‐Cl and 24 g of DMF (4 wt.%) was magnetically stirred at 60 °C until Cell‐Cl was completely dissolved. Subsequently, 3.43 mL of tributylphosphine was added, and the reaction was carried out for 48 h. When the reaction was finished, dialysis was performed using a dialysis tube (7000 Da) in DMF for 1 week to remove the unreacted reagents and then in water for 1 week to remove DMF. Finally, the solid Cell‐P^+^ was obtained by freeze‐drying.

### Cellulose‐Based Coating Lotion

0.1 g of Cell‐P^+^ was dissolved in 7 g of water, followed by adding 0.05 g of MMT. The mixture was stirred magnetically at room temperature until homogeneous and then dispersed ultrasonically for 40 min. Subsequently, the solution was added to 100 g of CMG (1.0 wt.%) and stirred magnetically at room temperature for 1 h. Finally, 0.025 g of Vc was dispersed into the solution and stirred for 10 min to prepare the cellulose‐based coating lotion.

### Fruit Coating by Spray or Dipping

Cellulose‐based barrier coating lotion can be applied to fruit surfaces through dipping or spraying methods. Dipping involves immersing the fruits (e.g., strawberries, Shine Muscat) in the coating lotion for 20 s to ensure thorough impregnation and then dried in the air for 5 min. At this point, the coating film was not completely dry (as depicted in Figure S9, Supporting Information). Subsequently, a second dipping in the coating lotion was conducted for 20 s. Afterward, the fruits were left to air dry for at least 60 min until the coating film was completely dry, a perfect coating on the fruits can be obtained (Figure 6a; Figures S10 and S17, Supporting Information). In the spraying method, the coating lotion was evenly sprayed onto the fruit's surface using an air‐blowing sprayer (distance, 5 cm). After 5 min of hanging and drying, a second layer was sprayed, and the fruit is dried in air. (The image accompanying this article depicts a sample that has been dip‐coated). The fruits were stored at an ambient temperature of 15 to 26 °C and an average relative humidity of 35% RH.

### Self‐Standing Films of the Coating Material

Self‐standing coating films are prepared to test the coating's oxygen permeability, carbon dioxide permeability, and water vapor transmittance rate and evaluate the coating lotion's mechanical properties. First, CMG and MMT in a certain ratio (Tables S1 and S2, Supporting Information) were thoroughly mixed using a magnetic stirring apparatus bar for at least 30 min. Then, the solution was filtered with a Buchner funnel. The residue was dried under a vacuum to obtain a self‐standing film (thickness,70 µm). The effect of water evaporation during the drying process at temperatures from room temperature to 80 °C can be dismissed (Figures S12 and S13, Supporting Information).

### Removal of Coating via H_2_O

To better visualize the cleaning process of the cellulose‐based barrier coating, the coating lotion was stained with methyl blue. Then, a quartz sheet was immersed in the coating lotion for 20 s and dried in the air for 5 min. The coating process was repeated to simulate the coating process on the fruits. Finally, the coating film on the quartz sheet was removed in water by ultrasonic cleaning for 30 s.

### Oxygen Permeability

The oxygen permeability (OP) of the self‐standing films was measured according to GB/T1038‐2000 using the Classic 216 differential pressure gas permeameter (23 °C, 0% RH), and the test area was 4.9 cm^2^ (duplicated samples, *n* = 3).

### Carbon Dioxide Permeability

The carbon dioxide permeability of the self‐standing films was measured according to GB/T1038.1‐2022 using the VAC‐V2 differential pressure gas permeameter (23 °C, 0% RH), and the test area was 38 cm^2^ (duplicated samples, *n* = 3).

### Water Vapor Transmission Rates

The water vapor transmission rates (WVTR) of the self‐standing films were measured using a water vapor test system (PERME W3/060, the cup method, 23 ± 2 °C, and 50 ± 2% RH), and the test area was 33.18 cm^2^ (duplicated samples, *n* = 3).

### Spreading Behavior of the Coating Lotion on the Fruits

The spreading behavior of coating lotion compacting on the fruit during spraying was recorded by a high‐speed camera (Optronis CP70‐1HS‐M‐1900‐RT, the frame rate, 5392 fps; the exposure time, 176 µs). The droplets were extruded slowly from a syringe needle (30 G, inner diameter 0.16 mm, outer diameter 0.31 mm) at a fixed height (8 cm) through a syringe pump to ensure the initial speed of the droplets was close to 0. The inner diameter of the syringe needle controlled the size of the droplet. Each sample was repeated three times.

### Antibacterial Activity Test

1. Preparation of the diluted bacteria suspension: A single colony of *E. coli* (or *S. aureus*) from LB agar plates was cultured in 10–15 µL of LB liquid medium at 37 °C (200 rpm) for 10–12 h. The bacterial suspension in the culture medium was centrifuged (7100 rpm, 2 min), and the obtained precipitate was washed three times with PBS (pH 7.4). Then, the bacterial suspension was diluted with PBS to achieve the desired bacterial concentration or optical density (OD) at 600 nm, specifically, OD_600_ (*E. coli*) = 1.0; OD_600_ (*S. aureus*) = 0.6.
2. Plate spreading and colony counting: First, 20 µL of the prepared *E. coli* (or *S. aureus*) suspension was respectively co‐cultured with 80 µL of PBS, CMG, CMG/MMT/Vc, coating lotion (1.0 wt.%) in the dark for 30 min. Then, the *E. coli* suspension for each test condition was serially diluted 10^4^‐fold with PBS. Finally, 100 µL of the above bacterial diluent was evenly coated on the LB agar plate and cultured in an incubator at 37 °C for 14–16 h until obvious colony‐forming units (CFUs) appeared on the medium (duplicated samples, n = 3). All experiments were repeated three times. The following formula calculated the inhibition rate:

(1) $$\mathrm{Inhibition}\;\mathrm{ratio}\left(\%\right)=\left(1-\frac{A}{A_{0}}\right)\times 100\%$$

where *A*_0_ is the CFU of PBS groups, and A is the CFU of experimental groups (CMG, CMG/MMT/Vc, Coating lotion) that were kept in the dark.

### Antioxidant Capacity Test

The antioxidant capacity of the coating lotion was evaluated by 1, 1‐diphenyl‐2‐trinitrohydrazine (DPPH) free radical scavenging rate. 2 mL of CMG, CMG/MMT/Cell‐P^+^, coating lotion, Vc were mixed with 2 mL of DPPH ethanol solution (0.1 mm), respectively. After incubation in the dark for 30 min, the absorbance (Abs) of the solution at 517 nm was measured by UV–vis spectrophotometer (duplicated samples, *n* = 4). The following formula calculated the DPPH scavenging rate:

(2) $$\mathrm{DPPH}\;\mathrm{scavenging}\;\mathrm{rate}\left(\%\right)=\left(1-\frac{A}{A_{0}}\right)\times 100\%$$

where *A*_0_ is the absorbance at 517 nm of the DPPH solution; *A* is the absorbance at 517 nm of the DPPH solution mixed with the sample lotion.

### Cytotoxicity Test

The cytotoxicity of coating lotion toward GES cells (human gastric mucosal epithelial cell line) was assessed using CCK‐8 assay. The GES cells (1×10^4^ cells per well) were cultured in DMEM medium (containing 10% FBS and 1% P/S solution) with a 96‐well microtiter plate and incubated at 37 °C in 5% CO_2_ atmosphere for 24 h. The coating lotion was diluted with DMEM in a concentration gradient (100, 10, 1, 0.1, 0.01 µg mL^−1^). Then, the medium was replaced with the dilution series of coating lotion and incubated at 37 °C in 5% CO_2_ for 24 h (duplicated samples, *n* = 4). The medium was sucked out, and 100 µL of fresh DMEM medium (containing 10% FBS and 1% P/S solution) and 10 µL of CCK‐8 reagent were added to further incubate the cell in the dark for 3 h. The absorbance (Abs) at 450 nm of each well is recorded with a microplate reader (CLARIOstar Plus, BMG Labtech). DMEM medium was set as the control group. Cell viability ratio (%) is calculated using the following formula:

(3) $$\mathrm{Cell}\;\mathrm{viability}\;\mathrm{ratio}\left(\%\right)=\mathrm{Ab}s_{\mathrm{sample}}/\mathrm{Ab}s_{\mathrm{control}}\;\times 100\%$$

### Dual‐Fluorescence Cell Viability Assay

Acridine orange (AO) and propidium iodide (PI) were used to determine the double fluorescence activity to further evaluate the compatibility of the coating lotion to GES cells. First, GES cells (2×10^4^ cells per well) were cultured in DMEM medium (containing 10% FBS and 1% P/S solution) with a 24‐well plate and incubated at 37 °C in 5% CO_2_ atmosphere for 24 h (duplicated samples, *n* = 4). Then, the medium was replaced with different concentrations of coating lotion (100, 10, 1, 0.1, 0.01 µg mL^−1^) and incubated at 37 °C in 5% CO_2_ for 24 h. The medium was sucked out, and 50 µL fresh DMEM medium (containing 10% FBS and 1% P/S solution) and 50 µL AO/PI staining solution were added for dark incubation for 15 min. Live/dead GES cells were observed using an inverted fluorescence microscope (Nikon Ti2‐U). DMEM medium was set as the control group.

### Mechanical Property Test

Mechanical properties of the Cellulose/MMT films were evaluated using a tensile testing instrument (Instron 3365, INSTRON). The samples were cut into 50×10 mm^2^ rectangular shapes for each group. The load cell was set at 2 kN, and the crosshead speed was 2 mm min^−1^ (duplicated samples, *n* = 5).

### Evaluation of Storage Quality of Fruit

The calculation of the fruit's weight loss was determined by subtracting the weight of the first day of coating (*W_0_*) from the subsequent days of coating (*W_t_*) using the following formula:

(4) $$\mathrm{Weight}\;\mathrm{loss}\left(\%\right)=\left(1-\frac{W_{t}}{W_{0}}\right)\;\times 100\%$$

### Instruments

^1^H NMR spectra were acquired using an NMR spectrometer (AVANCE 400 m, Bruker) at room temperature with tetramethylsilane (TMS) as the reference compound in DMSO. Fourier Transform Infrared (FTIR) spectra were recorded using a Nicolet 6700 spectrometer (Thermal Fisher) for analysis. UV–vis spectra were investigated using a UV 2600 spectrometer (Shimadzu) integrated with an ISR‐2600 Plus sphere. The zeta potential of the sample was determined using a Zetasizer (Nano ZS ZEN3600, Malvern). The micromorphology of the nanocomposite film was examined using a scanning electron microscope (SEM, SU8020, Hitachi). The images of agar plates were captured using a gel imaging system (GenoSens 2200, Clinx Science Instruments, Shanghai). The spreading process of the droplets on the fruit surface was recorded using a high‐speed camera (CP70‐1HS‐M‐1900‐RT, Optronics). The water contact angle (WCA) was determined using a droplet size analyzer (DSA‐100; Krüss, Germany). The X‐ray diffraction pattern was recorded using an X‐ray diffractometer (PANalytical, Netherlands) with a wavelength of the radiation source of 1.54 Å (Cu Kα) in reflection mode for 2*θ* 2–40° at a speed of 5° min^−1^. The size distribution was measured using a laser diffraction particle size analyzer (Bettersizer 2600).

### Statistical Analysis

All data are presented as mean ± standard deviation (SD) according to duplicated experiments at least three times. The two‐tailed t‐test was used to determine the statistical significance between two groups, and *p* < 0.05 was considered statistically significant. *p* values for two tailed tests: ^*^<0.05, ^**^<0.01, ^***^<0.001, ^****^<0.0001.

## Conflict of Interest

The authors declare no conflict of interest.

## Supporting information



Supporting Information

## Data Availability

The data that support the findings of this study are available from the corresponding author upon reasonable request.
