# Supplementary material for: Cellulose‐Based Transparent Edible Antibacterial Oxygen‐Barrier Coating for Long‐Term Fruit Preservation
Source: Adv Sci (Weinh). 2024 Nov 13;11(48):2409560. doi: 10.1002/advs.202409560 (PMC11672319; doi:10.1002/advs.202409560)
Supplement: Supplementary file 1 — Supporting Information [file ADVS-11-2409560-s001.docx]

**Supporting Information**

**Cellulose-based transparent edible antibacterial oxygen-barrier coating for long-term fruit preservation**

Yuqian Cui ^a, b^, Yixiu Cheng ^a, b^, Zhan Xu ^a, b^, Bingchun Li ^a, b^, Weiguo Tian ^a,*^, Jun Zhang ^a, b, *^

^a^ *Beijing National Laboratory for Molecular Sciences, CAS Key Laboratory of Engineering Plastics, Institute of Chemistry Chinese Academy of Sciences (CAS), Beijing 100190, China;*

^b^ *University of Chinese Academy of Sciences, Beijing 100049, China;*

^*^ *E-mail:* [*wgtian@iccas.ac.cn*](mailto:wgtian@iccas.ac.cn)*; jzhang@iccas.ac.cn*

**Content**

1. **Supplementary Figures :** Figure S1 to S18
2. **Supplementary Tables :** Table S1 to S3
3. **Supplementary discussions**
4. **References**
5. **Supplementary Figures**


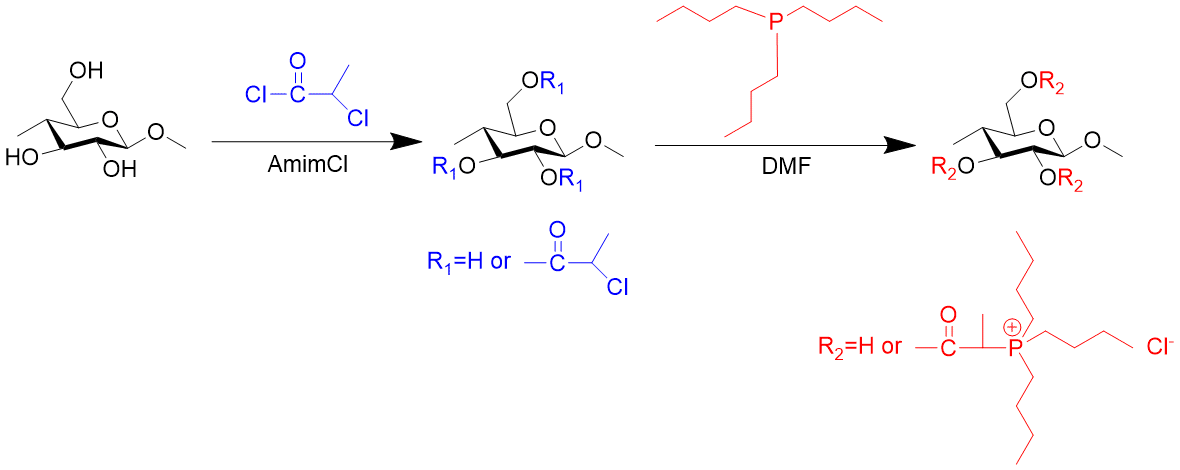


**Figure S1** Synthesis route of the cationic cellulose derivative Cell-P^+^.


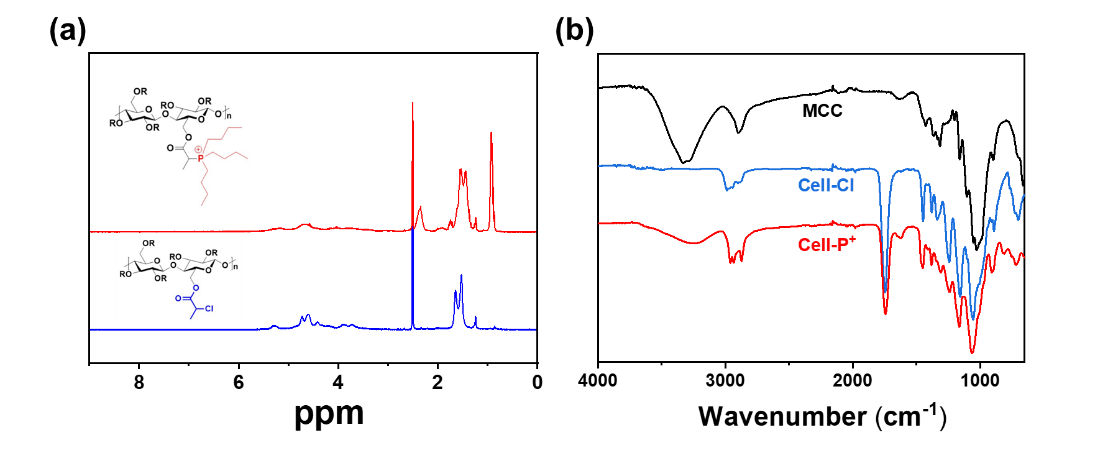


**Figure S2** ^1^H NMR and FTIR spectra of the cationic cellulose derivative (Cell-P^+^) and its precursor (Cell-Cl).

**
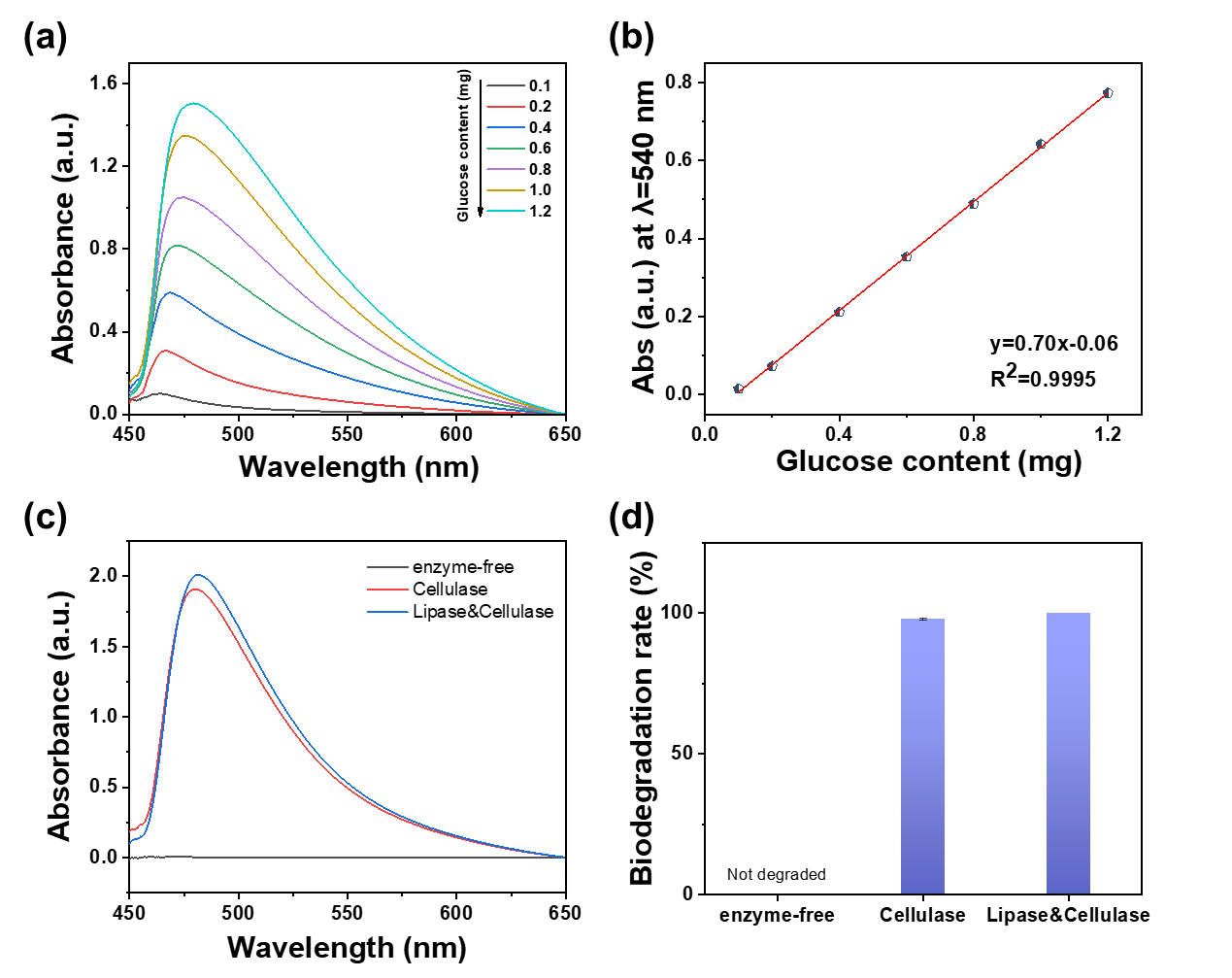
**

**Figure S3** **(a)** UV-Vis absorption spectra of glucose at different content **(b)** Content-absorbance standard curve of glucose at 540 nm **(c)(d)** Biodegradation rate of Cell-P^+^ treated with lipase-cellulase (n = 3).


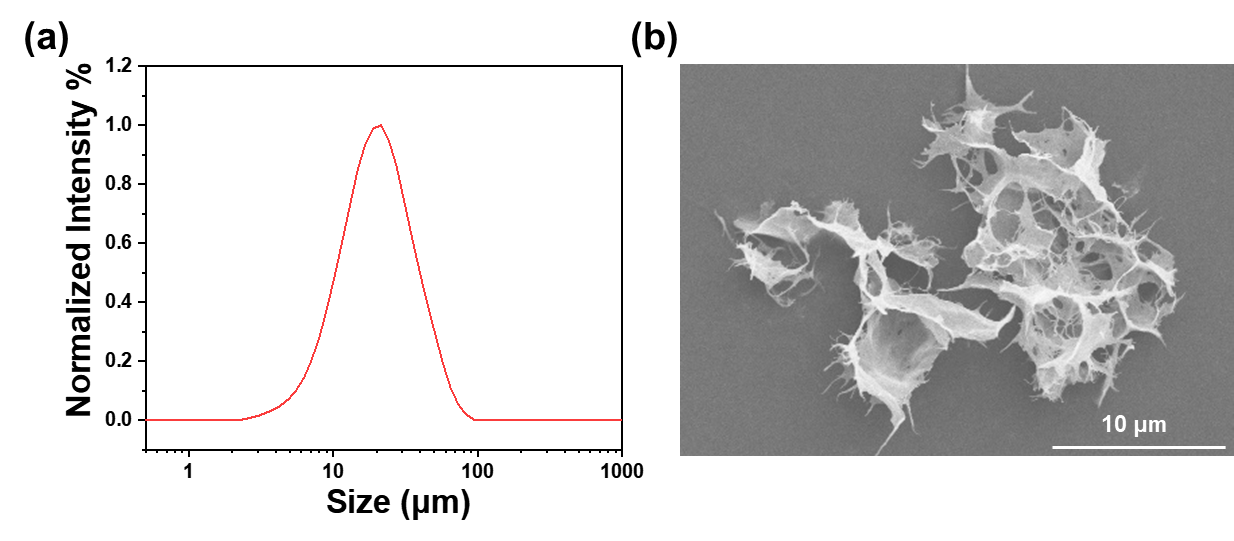


**Figure S4 (a)** Size distribution and **(b)** micromorphology of CMG

**Figure S5** XRD analysis of CMG, MMT, and MMT/Cell-P^+^.


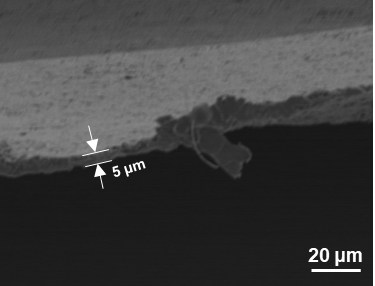


**Figure S6** Cross-sectional morphology of coated film.

**
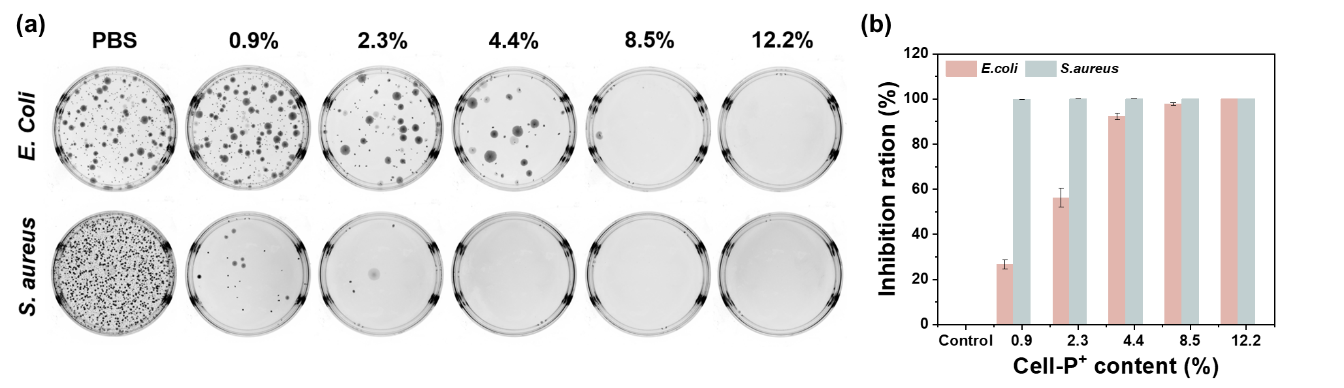
**

**Figure S7 (a)** images of the bacterial colonies and **(b)** the related inhibition ratios of *E. coli* and *S. aureus* incubated with coating lotion (1.0 wt.%) containing different Cell-P^+^ contents in the dark for 30 min (n = 3).


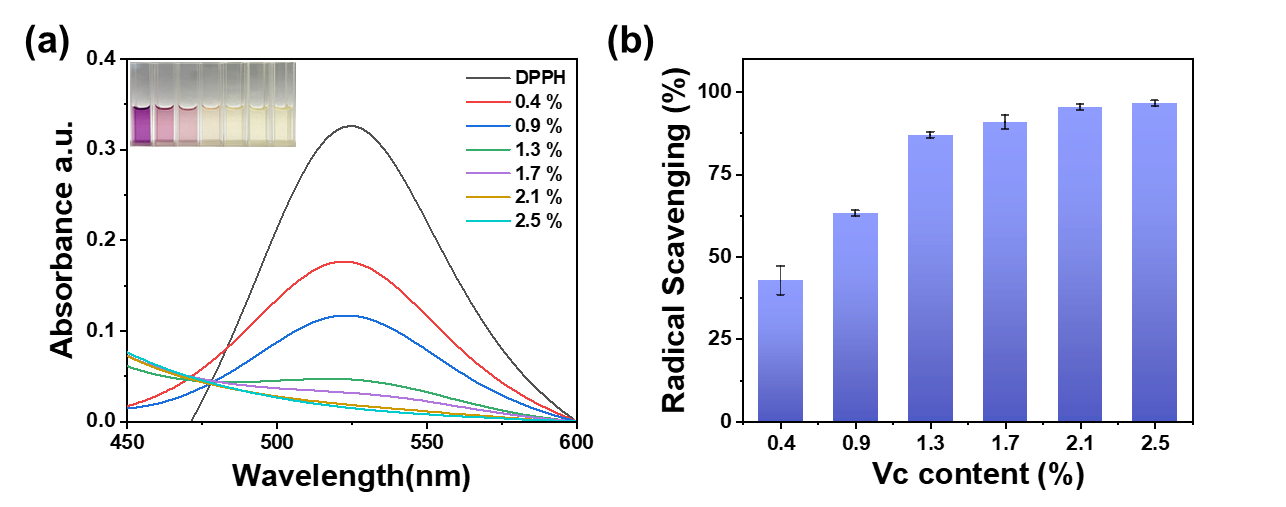


**Figure S8 (a)** UV-Vis spectra and images (inset), **(b)** DPPH radical scavenging activity of coating lotion (1.0 wt.%) with different Vc contents (n = 4).


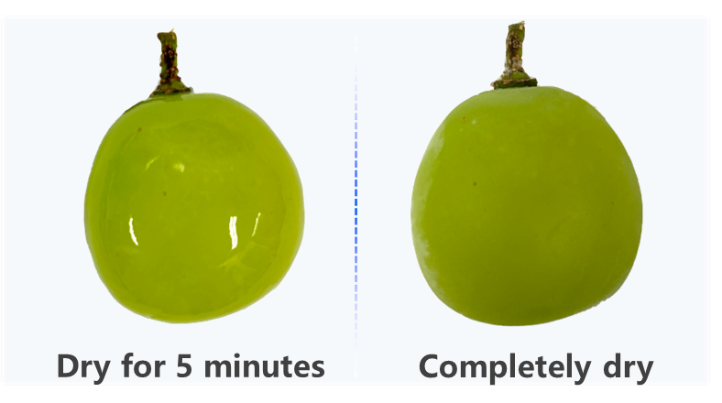


**Figure S9** Images of the coated Shine Muscat dried in air for different periods of time.


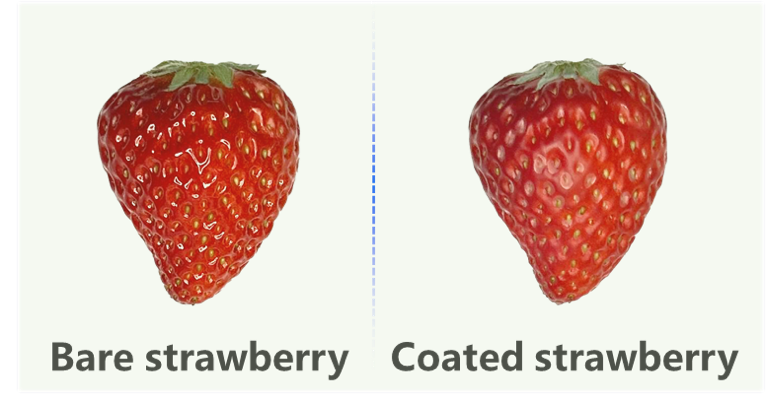


**Figure S10** Digital images of the bare strawberries and coated strawberries.


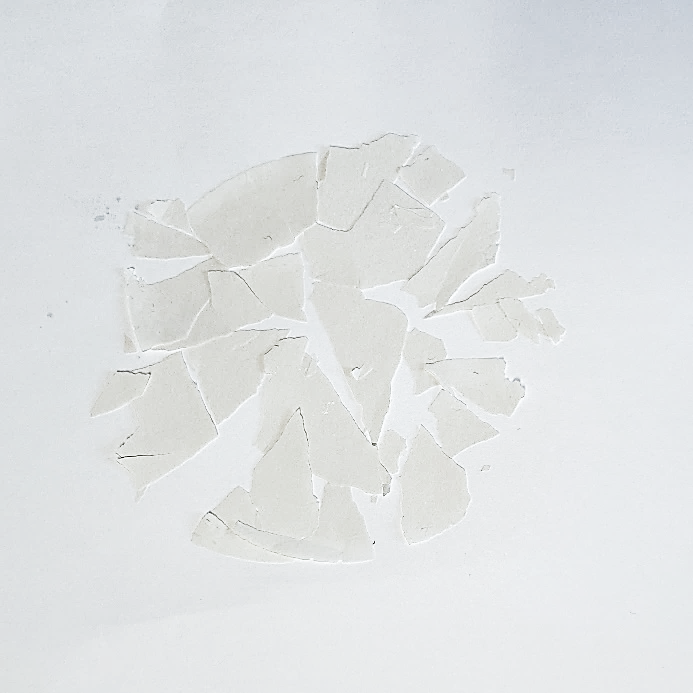


**Figure S11** Fragments of the fragile MMT film obtained from the MMT suspension.

**Figure S12** Density densities of the coating films dried through different methods at different temperatures.


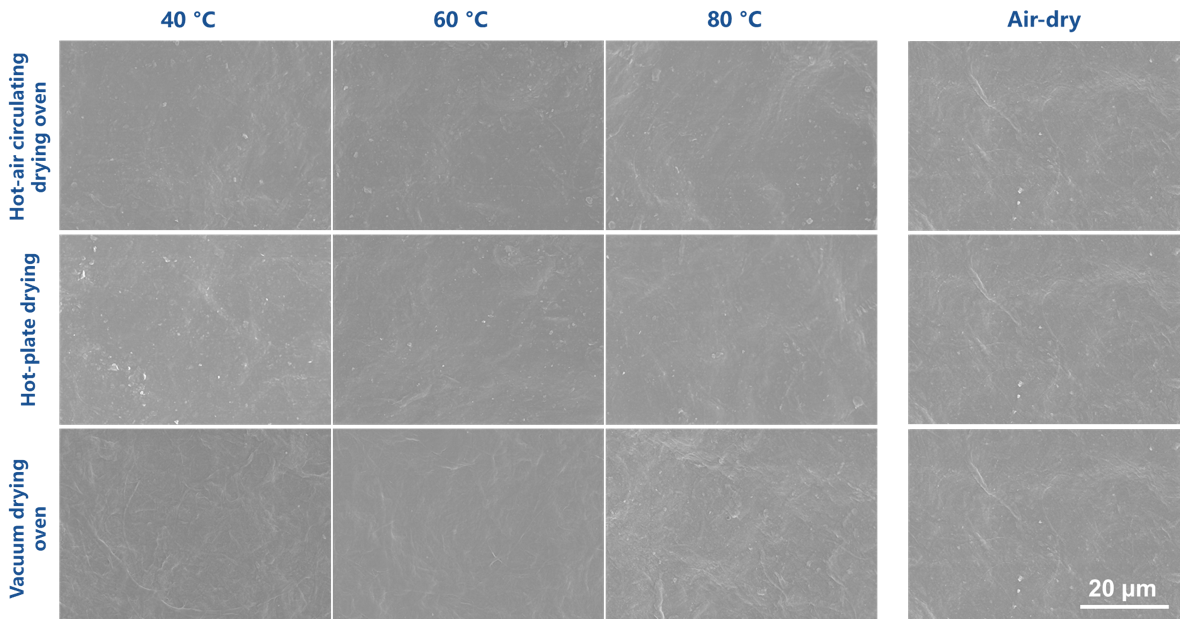


**Figure S13** Surface micro-morphologies of the coating films dried through different methods at different temperatures.


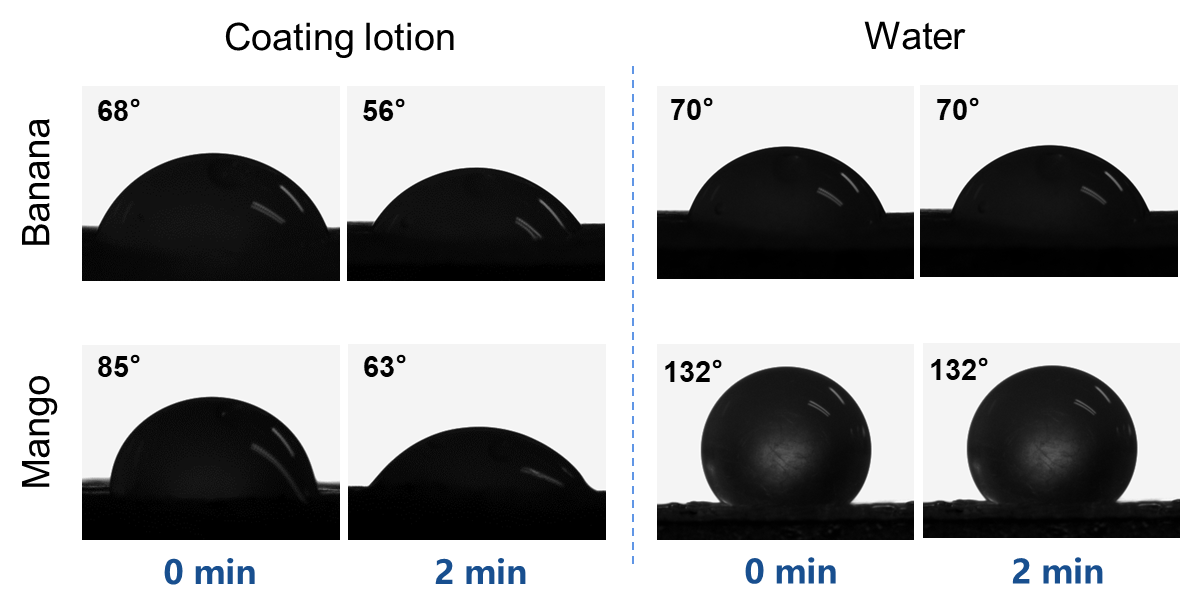


**Figure S14** Contact angle analysis of the coating lotion and water on different fruit surfaces.


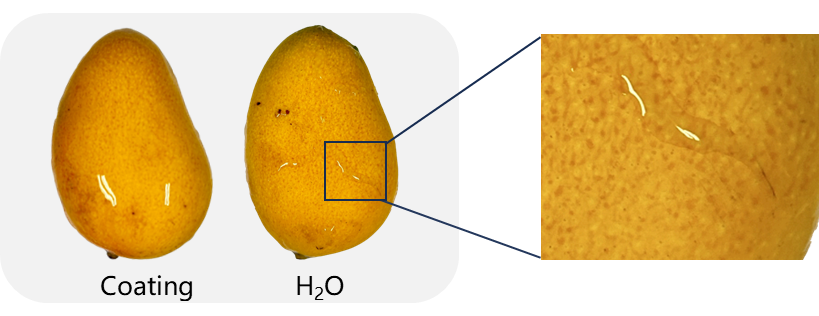


**Figure S15** Comparison of the spreading behaviors between coating lotion and water on the surface of a mango.


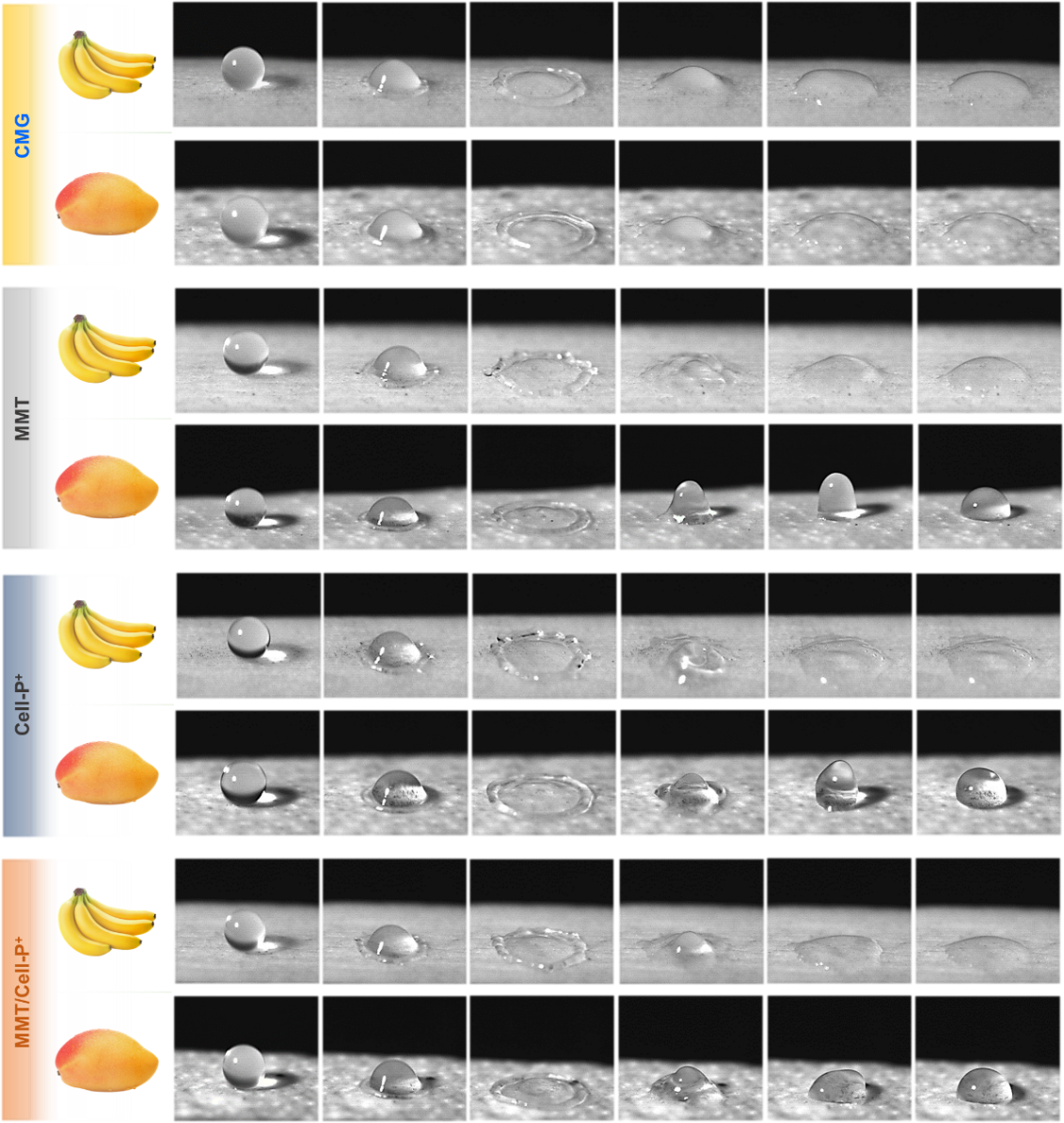


**Figure S16** Spreading behavior of various droplets (CMG, MMT, Cell-P^+^, MMT/Cell-P^+^) compacting on different fruit surfaces (banana and mango).

**
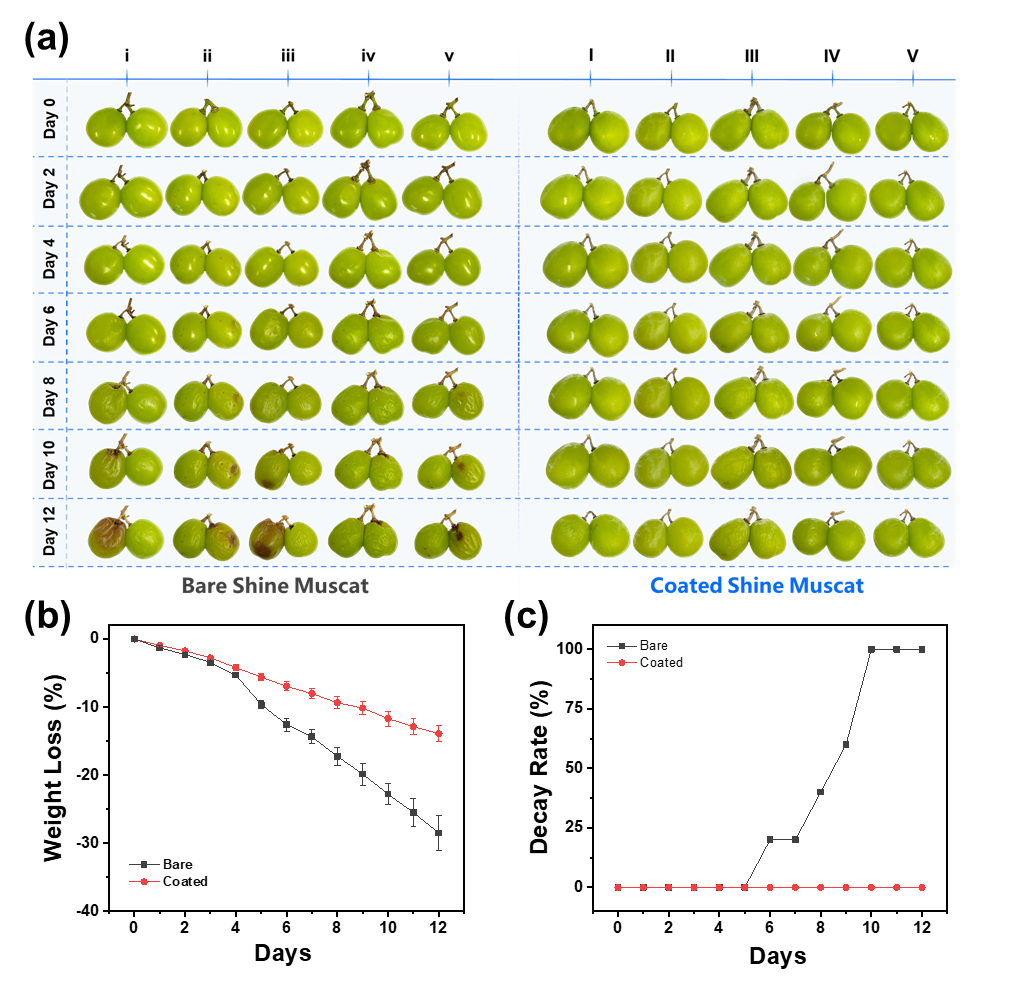
**

**Figure S17 (a)** Time-lapse photographs of bare and coated Shine Muscat. **(b)(c)** Weight loss and decay rate of Shine Muscat during storage.


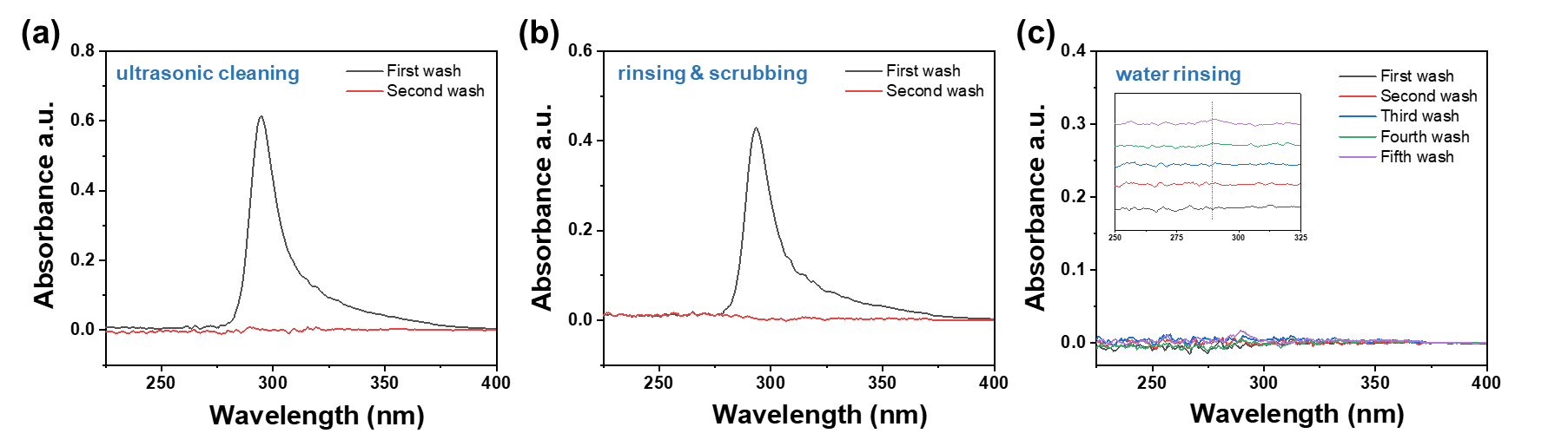


**Figure S18** UV-Vis detection of the potential residual coating material on fruits (Shine Muscat) washed by different cleaning methods.

1. **Supplementary Tables**

**Table S1** Components and their ratios in the films for oxygen, carbon dioxide, and water vapor transmission rate tests.

| **Sample** | **CMG**  (1.0 wt.%, g) | **MMT**  (1.0 wt.%, g) | **CMG/MMT** | **Thickness**  (μm) |
| --- | --- | --- | --- | --- |
| 100%CMG/0%MMT | 50 | 0 | 50/0 | 55 |
| 98.0%CMG/2.0%MMT | 50 | 1 | 50/1 | 56 |
| 95.2%CMG/4.8%MMT | 50 | 2.5 | 50/2.5 | 56 |
| 90.9%CMG/9.1%MMT | 50 | 5 | 50/5 | 59 |
| 87.0%CMG/13.0%MMT | 50 | 7.5 | 50/7.5 | 61 |

**Table S2** Components and their ratios in the films for mechanical test.

| **Sample** | **CMG**  (1.0 wt.%, g) | **MMT**  (1.0 wt.%, g) | **CMG/MMT** | **Thickness**  (μm) |
| --- | --- | --- | --- | --- |
| 100%CMG/0%MMT | 50 | 0 | 50/0 | 55 |
| 98.0%CMG/2.0%MMT | 50 | 1 | 50/1 | 56 |
| 95.2%CMG/4.8%MMT | 50 | 2.5 | 50/2.5 | 56 |
| 90.9%CMG/9.1%MMT | 50 | 5 | 50/5 | 59 |
| 87.0%CMG/13.0%MMT | 50 | 7.5 | 50/7.5 | 61 |
| 83.3%CMG/16.7%MMT | 50 | 10 | 50/10 | 62 |
| 76.9%CMG/23.1%MMT | 50 | 15 | 50/15 | 67 |
| 71.4%CMG/28.6%MMT | 50 | 20 | 50/20 | 71 |
| 100%MMT/0%CMG | 0 | 60 | 0/60 | / |
| 95.2%MMT/4.8%CMG | 3 | 60 | 3/60 | 70 |
| 90.9%MMT/9.1%CMG | 6 | 60 | 6/60 | 71 |
| 87.0%MMT/13.0%CMG | 9 | 60 | 9/60 | 77 |
| 83.3%MMT/16.7%CMG | 12 | 60 | 12/60 | 78 |
| 71.4%MMT/28.6%CMG | 24 | 60 | 24/60 | 86 |
| 62.5%MMT/37.5%CMG | 36 | 60 | 36/60 | 106 |

Note: MMT cannot form self-standing film, and the film thickness data is not available.

**Table S3** pH of different samples

| Sample | H_2_O | CMG | MMT | Cell-P^+^ | Lotion |
| --- | --- | --- | --- | --- | --- |
| pH | 6.23 | 6.49 | 6.30 | 3.03 | 2.98 |

1. **Supplementary discussions**

***Synthesis of Cell-P^+^.*** The synthesis of antibacterial cationic cellulose derivatives (Cell-P^+^) involved a two-step process as depicted in Figure S1. Initially, cellulose chloropropionate (Cell-Cl) was produced through the esterification of the hydroxyl groups on cellulose using 2-chloropropionyl chloride. Subsequently, Cell-P^+^ was synthesized through nucleophilic substitution of Cell-Cl with tributylphosphine.

***Chemical structures of Cell-Cl and Cell-P^+^.*** The chemical structures of Cell-Cl and Cell-P^+^ were authenticated through ^1^H NMR (Figure S2a) and FTIR (Figure S2b) analyses. In the ^1^H NMR spectrum of Cell-Cl, a prominent peak at 1.6 ppm was attributed to the H of -CH_3_ in 2-chloropropionyl chloride, while the H of -CH_2_- in the cellulose backbone and 2-chloropropionyl chloride was observed in the range of 2.5-5.5 ppm. The DS (degree of substitution) of -Cl was calculated to be 3.0. Furthermore, ^1^H NMR spectrum of Cell-P^+^ exhibited a peak at 0.9 ppm for the H of -CH_3_ in tributylphosphine, along with peaks at 1.4 ppm and 2.4 ppm for the H of -CH_2_-. The DS of tributylphosphine was determined to be 2.0 based on the peak area calculation. The FTIR analysis identified a stretching vibration peak at 1740 cm^-1^ for C=O, a stretching vibration peak at 1000 cm^-1^ for C-O-C, and a stretching vibration peak near 2900 cm^-1^ for C-H in the -CH_3_, coinciding with the ^1^H NMR results and confirming the successful synthesis of Cell-P^+^.

***Biodegradability of Cell-P^+^.***  Given that Cell-P^+^ is water-soluble, it is challenging to assess its biodegradability using the conventional compost method. Therefore, the enzymatic degradability of Cell-P^+^ was investigated using the colorimetric method of 3,5-dinitrosalicylic acid ^[1, 2]^ as follows: First, 0.04 g Cell-P^+^ was dissolved in 5 ml sodium hydrogen phosphate/citric acid buffer at pH=7.2. Then 6 mg lipase was added and the solution was incubated in a shaker at 37 ℃ for 3 days. Subsequently, citric acid was added to adjust the pH to 4.8 and 0.64 mg cellulase was added. Afterward, the mixture was incubated in a shaker at 50 ℃ for 2 days. The absorbance of 3,5-dinitrosalicylic acid at 540 nm was measured utilizing a UV spectrophotometer, and the reducing sugar content was calculated employing the glucose content-absorbance standard curve (Fig. S3a and 3b). The enzyme degradation rate was determined as the ratio of the total mass of reducing sugar to the sample mass. The experimental results demonstrated that Cell-P^+^ could be completely degraded to glucose under the synergistic action of lipase and cellulase, with a degradation rate of 100 %, and nearly completely degraded under the action of cellulase alone, with a degradation rate of 97.8 %. conclusively, Cell-P^+^ exhibits favorable biodegradability and it is unlikely to cause any adverse environmental effects.

***Preparation of CMG.*** 4 g of ground eucalyptus pulp is soaked with 96 g of AmimCl in a three-neck round flask. The mixture is heated to 80 ^o^C and continuously stirred under a vacuum for at least 2 h until the pulp is thoroughly dissolved. Then, the solution is poured into ethanol for gelation. The cellulose gel is washed using deionized water several times to remove AmimCl and ethanol. Afterward, shatter the gel with a blender for 3 min. The shattered cellulose hydrogel is filtered, and water is added to re-suspend the hydrogel particles (total weight, 400 g; solid content of cellulose, 1 wt.%). The suspension is treated using a high-pressure homogenizer (APV2000) at 600 bar for 3 cycles, resulting in a homogeneous CMG suspension. The size distribution and micromorphology of CMG are shown in Figure S4.

***Optimal addition of Cell-P^+^.*** The results from Figure S7 indicate that the antibacterial activity improved as the amount of Cell-P^+^ increased. For *E. coli*, the antibacterial rate exceeded 98 % when 8.5 % Cell-P^+^ was added and reached 100 % when 12.2 % was added. For *S. aureus*, the antibacterial rate was over 99 % at various levels of Cell-P^+^ addition and reached 100 % at 4.4 %. Based on these findings, we concluded that the optimal amount of Cell-P^+^ to add to the lotion is 8.5 %.

***Optimal addition of Vc.*** As illustrated in Figure S8, the gradual increase in Vc content resulted in the progressive fading of the purplish-red DPPH ethanol solution until it transformed into a transparent light-yellow solution. Concurrently, the corresponding UV-vis absorption peak at 517 nm gradually diminished until it completely disappeared. Figure S8b demonstrates that the DPPH radical scavenging rate increased proportionally with the addition of Vc, reaching over 95 % at a Vc addition of 2.1 %. However, further increases in Vc content did not yield significant improvements in the antioxidant properties of the lotion. Consequently, the optimal Vc addition in this study was determined to be 2.1 %.

***Effect of drying process on the density and surface characteristics of coating films.*** Different composite films were obtained through various drying methods at different temperatures. Considering that the drying temperature should not exceed a certain threshold during fruit preservation, the maximum drying temperature was established at 80 °C. As illustrated in Figure S12, there is no significant difference between the densities of the films dried at different temperatures. The films dried at 60 °C have almost the same density as those dried in air. As shown in the SEM images (Figure S13), the drying methods and temperatures did not demonstrate significant effects on the surface micromorphology of the films within the temperature range from room temperature to 80 °C. Therefore, we believe that the effect of water evaporation during the drying process at temperatures from room temperature to 80 °C can be dismissed.

***UV-Vis analysis of the potential residual coating material on the washed fruit.*** We used a more sensitive test to evaluate the retention of coatings after washing. The sensitivity of UV-Vis spectroscopic detectors is considerably greater than that of the FTIR method. UV-Vis spectroscopy is usually employed as the HPLC detector for analyzing residual substances. Herein, Vc, a main component in the coating film, is used as an indicator to analyze the residual coating materials on the washed fruits via the UV-Vis spectroscopic method. Following ultrasound cleaning (Fig. S18a) and manual scrubbing (Fig. S18b), a distinct characteristic peak of Vc at 290 nm was observed in the wash water, indicating effective removal of the coating from the fruit. After the second round of the cleaning process, the peak at 290 nm disappeared, confirming the absence of Vc in the water and suggesting that the coating was fully removed after the first cleaning. In contrast, only water soaking or rinsing (Figure S18c) cannot effectively remove the coating film from the fruit, even several times. The above results demonstrate that the adhesion between the coating film and the fruit is strong enough to prevent water soaking. However, the coating film can be effectively removed by ultrasonic or scrubbing methods before consumption.

**References**

[1] Xu, R.; Yin, C.; Zhang, J.; Zhou, Y.; Mi, Q.; Zhang, J. *ACS Sustainable Chem. Eng.* **2022**, 10 (8), 2822–2829.

[2] Chua, M.; Chan, K.; Hocking, T. J.; Williams, P. A.; Perry C. J., Baldwin, T. C.; *Carbohydr. Polym.* **2012**, 87 (3), 2202–2210.
